# Molecular Insights of p47^phox^ Phosphorylation Dynamics in the Regulation of NADPH Oxidase Activation and Superoxide Production*

**DOI:** 10.1074/jbc.M114.561159

**Published:** 2014-06-26

**Authors:** Daniel N. Meijles, Lampson M. Fan, Brendan J. Howlin, Jian-Mei Li

**Affiliations:** From the ‡ Faculty of Health and Medical Science, University of Surrey, Surrey GU2 7XH, United Kingdom,; the ¶ John Radcliffe Hospital, University of Oxford, Oxford OX3 9DU, United Kingdom, and; the § Faculty of Engineering and Physical Sciences, University of Surrey, Surrey GU2 7XH, United Kingdom

**Keywords:** Computer Modeling, Endothelial Cell, Molecular Docking, Molecular Dynamics, NADPH Oxidase, Phosphorylation, Site-directed Mutagenesis

## Abstract

Phagocyte superoxide production by a multicomponent NADPH oxidase is important in host defense against microbial invasion. However inappropriate NADPH oxidase activation causes inflammation. Endothelial cells express NADPH oxidase and endothelial oxidative stress due to prolonged NADPH oxidase activation predisposes many diseases. Discovering the mechanism of NADPH oxidase activation is essential for developing novel treatment of these diseases. The p47^phox^ is a key regulatory subunit of NADPH oxidase; however, due to the lack of full protein structural information, the mechanistic insight of p47^phox^ phosphorylation in NADPH oxidase activation remains incomplete. Based on crystal structures of three functional domains, we generated a computational structural model of the full p47^phox^ protein. Using a combination of *in silico* phosphorylation, molecular dynamics simulation and protein/protein docking, we discovered that the C-terminal tail of p47^phox^ is critical for stabilizing its autoinhibited structure. Ser-379 phosphorylation disrupts H-bonds that link the C-terminal tail to the autoinhibitory region (AIR) and the tandem Src homology 3 (SH3) domains, allowing the AIR to undergo phosphorylation to expose the SH3 pocket for p22^phox^ binding. These findings were confirmed by site-directed mutagenesis and gene transfection of p47^phox−/−^ coronary microvascular cells. Compared with wild-type p47^phox^ cDNA transfected cells, the single mutation of S379A completely blocked p47^phox^ membrane translocation, binding to p22^phox^ and endothelial O_2_^⨪^ production in response to acute stimulation of PKC. p47^phox^ C-terminal tail plays a key role in stabilizing intramolecular interactions at rest. Ser-379 phosphorylation is a molecular switch which initiates p47^phox^ conformational changes and NADPH oxidase-dependent superoxide production by cells.

## Teaser

**Background:** p47^phox^ is a regulatory subunit of NADPH oxidase, which produces superoxide.

**Results:** We propose a molecular dynamics model of p47^phox^ phosphorylation and assembly with p22^phox^ in NADPH oxidase activation supported by biochemical experiments.

**Conclusion:** Ser-379 phosphorylation in the C-terminal tail is a molecular switch in p47^phox^ activation.

**Significance:** This report provides novel structural and mechanistic information of p47^phox^ activation.

## Introduction

NADPH oxidase is an O_2_^⨪^-generating enzyme expressed in a variety of mammalian cell types (1), and NADPH oxidase activation in phagocytes (oxidative burst) is essential for immune response against infection (2). However excessive and unabated reactive oxygen species (ROS)^2^ production by this enzyme causes chronic inflammation and multi-organ oxidative damage with severe consequences. Vascular endothelial cells also express constitutively NADPH oxidase, which generates consistently low levels of O_2_^⨪^ involved in redox-signaling under physiological conditions. However, under pathological conditions, NADPH oxidase is activated and excessive ROS production causes endothelial dysfunction, which is an early hallmark predisposing many diseases such as atherosclerosis and hypertension (1, 3). Insight into the regulatory mechanism of NADPH oxidase activation is essential for developing novel therapeutic strategies to treat these oxidative stress-related cardiovascular diseases without affecting the neutrophil oxidative response to infection (4).

The NADPH oxidase is a multi-component enzyme, which consists of a cytochrome b_558_ containing a catalytic subunit Nox2 (also called gp91^phox^) and a p22^phox^, and several cytosolic regulatory subunits *i.e.* p47^phox^, p67^phox^, p40^phox^, and Rac (5, 6). The phosphorylation of p47^phox^ (a major regulatory subunit) has been recognized to be a prerequisite of NADPH oxidase activation (7–12). The p47^phox^ consists of an N-terminal PX-domain which interacts with cell membrane phosphoinositides; two tandem Src homology 3 (SH3) domains forming a super-SH3 (sSH3) binding groove for binding to the proline rich region (PRR) of p22^phox^; a polybasic auto-inhibitory region (AIR), and a C-terminal PRR domain for interaction with other NADPH oxidase subunits (1, 6, 13). In the resting state, the sSH3 groove is masked by the AIR to keep p47^phox^ in its autoinhibited conformation. Phosphorylation of serine residues *i.e.* Ser-303–304, Ser-310, Ser-315, Ser-320, and Ser-328 within the AIR results in AIR destabilization, which exposes the sSH3 groove for p22^phox^ to bind and to activate NADPH oxidase (14, 15). However, p47^phox^ has several serine phosphorylation sites outside the AIR toward the C terminus (14), yet their phosphorylation and the position of the C terminus tail (residues 341–390) in the p47^phox^ global conformation remains unclear.

It had been shown previously that phosphorylation of Ser-379 is a key step required for p47^phox^ membrane translocation and interactions with other proteins, and a single substitution of Ser-379 almost abolished leukocyte NADPH oxidase activity (16, 17), and TNFα-induced NADPH oxidase-dependent O_2_^⨪^ production in endothelial cells (18). However, the molecular mechanism of how a single serine (Ser-379) phosphorylation can promote NADPH oxidase activation is unknown.

In the current study, we have generated, for the first time, an *in silico* model of the complete p47^phox^ protein structure and shown the importance of the C-terminal tail in stabilizing the p47^phox^ structure at rest. We have demonstrated step by step the phosphorylation-induced p47^phox^ conformational changes and protein/protein interactions with p22^phox^ by molecular dynamics. The *in silico* results were further confirmed by site-directed mutagenesis and gene transfection of p47^phox−/−^ cells. Our study has discovered a molecular switch in initiating p47^phox^ activation and revealed an important mechanism for how p47^phox^ becomes activated from partial to full opening of the sSH3 groove for p22^phox^ binding and O_2_^⨪^ production by NADPH oxidase.

## MATERIALS AND METHODS

### 

#### 

##### Generation of a Full p47^phox^ Protein Structural Model

The auto-inhibited p47^phox^ (1–390 amino acids) model was generated based on three available crystal structures *i.e.* the N-terminal PX domain (residues 1–141, PDB: 1KQ6); the p47^phox^ PRR domain (residues 359–390, PDB: 1K4U) (19); and the super-SH3 domain (sSH3) (residues 159–340, the 1NG2 crystallized structure) (14), which was kindly provided by Dr. Franca Fraternali, King's College London, UK. The missing linking segments (142-MKDGKSTATDITGPII-156 and 340-PGPQSPGSPLEEERQTQRSK-360) were generated using the web-based homology protein modeling server Phyre2 as described previously (20–22). Briefly, the short residue sequences were uploaded to the server, and underwent template identification and structure refinement. The subsequent Phyre2 models scored 50 and 90% structure confidences, respectively, which satisfied structural motifs predicted by SWISS-MODEL (23) and PHDsec (24). The generated Phyre2 linking peptides had 5 residue extensions at both the N- and C-terminal sides to superimpose with the corresponding ends of the protein crystal structures, and the protein backbones were joined together manually in molecular operating environment (MOE; Chemical Computing Group Inc., Canada). The final model of the full p47^phox^ protein (a.a.1–390) was constructed using the protein homology modeling function in MOE, and was refined by energy minimization using the AMBER99 force field as described previously (25).

##### Structural Analysis

The secondary structure, residue contacts and water affinities of the energy minimized models were analyzed using the protein geometry function in MOE. The stereochemical qualities of the models were assessed by using Ramachandran plot analysis and structural analysis using the protein report function in MOE. This searches for disallowed bond angles, bond lengths and side chain rotamers. There were no unacceptable deviations in the models with less than 2 outliers in the Ramachandran plot.

##### In Silico p47^phox^ Phosphorylation and Molecular Dynamics

The effect of phosphorylation-induced p47^phox^ conformational changes was investigated *in silico* by adding a phosphate group (PO_4_^3−^) to the side-chain of each serine residue of Ser-303, Ser-304, Ser-310, Ser-315, Ser-320, Ser-328, Ser-345, Ser-348, Ser-359, Ser-370, and Ser-379 using the molecular builder function of MOE. The phosphorylated p47^phox^ model then underwent energy minimization for structural refinement to correct any potential disallowed bond angles, bond lengths and unfavorable torsion angles following PO_4_^3−^ addition.

The p47^phox^ conformational changes associated with serine phosphorylation were studied by molecular dynamics (MD). Prior to MD simulation the protein was prepared by addition of missing hydrogen atoms which were unable to be located in low resolution protein x-ray structures. The hydrogen atoms were added at positions calculated using the Protonate 3-dimensional module of MOE. Next, the protein was solvated in a square-box of water, which was positioned randomly to occupy the volume using the solvate module in MOE. Finally, sodium ions were added to the simulation box to balance charges, and followed by energy minimization to remove any high energy interactions or van der Waals violations. The MD simulation was recorded every 0.5 picoseconds (ps) at a constant temperature of 310K for 500 ps, with a time step of 0.002 ps. The algorithm employed was the Nosé-Poincaré-Anderson with an NPT ensemble. The force field used was AMBER99 with a distance-dependent dielectric of 4 for the protein and 80 for the solvent. This resulted in a stable simulation after initial equilibration of the structure under calculated conditions. The simulation was considered to have been equilibrated after the first 100 ps.

The NPA Hamiltonian used in MOE is shown in Equations 1 and 2,

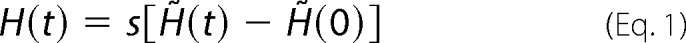


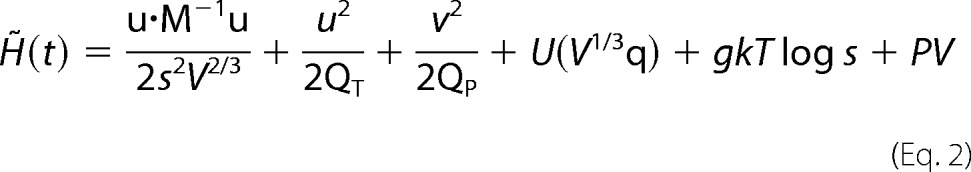
 where *g* is the number of degrees of freedom in the atomic system plus one if *V* is not fixed. The *s* coordinate is a time scaling coordinate used to enforce constant temperature. The quantity *H* is conserved, and the equations of motion can be derived by differentiating *H* with respect to the conjugate coordinates and momenta to obtain the equations of motion.

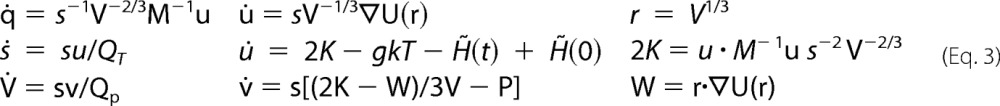
 To solve these equations, the symplectic, time-reversible Generalized Leapfrog Algorithm is used. A Hamiltonian, *H* (**p**,**q**) in which **q** are the coordinates and **p** the conjugate momenta at time *t* can be integrated through a time step *h* with Equations 4–6,

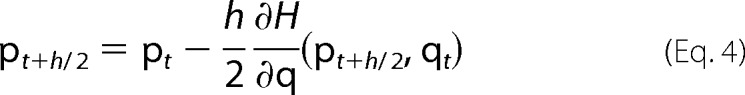


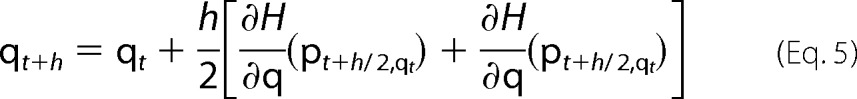


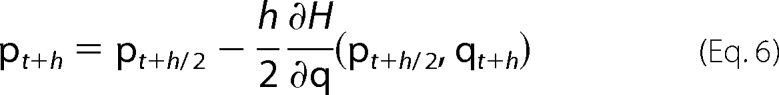
 which is a concatenation of the Symplectic Euler method with its adjoint (its reverse time analog) (see the MOE user manual).

##### Molecular Docking

Protein docking is a well-established technique for probing the proteome or interactome (26). Protein-protein interaction between the p47^phox^ sSH3 domain and the p22^phox^ PRR domain was examined by molecular docking using MOE. A 1.80Å resolution x-ray structure of the p22^phox^ (14) PRR was positioned near to the AIR region before and after phosphorylation and the molecular dynamics was re run for 500 ps to see if the p22^phox^ peptide would migrate into the p47^phox^ sSH3 binding groove. This dynamics simulation was followed by energy minimization to convergence. The resulting root mean squared deviation (RMSD) following MD when compared with the orientation of the crystallized p22^phox^ peptide was 1.676Å, and the peptide interacted successfully with the p47^phox^ in the sSH3 binding groove.

##### Site-directed Mutagenesis, Cell Culture, and Gene Transfection

The substitution of serine (S) by alanine (A) in human p47^phox^ cDNA (GenBank^TM^: AF330627.1) was performed exactly as described previously using the QuikChange Multi Site-directed Mutagenesis kit (Agilent Technologies) (18). Briefly, Wild-type p47^phox^ cDNA was cloned into pcDNA3.1/Zeovector (Invitrogen) and used for the mutagenesis. Mutated p47^phox^ cDNAs were then sequenced and cloned into *Escherichia coli*, DH5α (Invitrogen) (18). The plasmids were purified for gene transfection.

The p47^phox^ knock-out (KO) mice on a 129sv background were obtained from the European Mouse Mutant Archive, and backcrossed to C57BL/6J for 10 generations. All studies were performed in accordance with protocols approved by the Home Office under the Animals (Scientific Procedures) Act 1986 UK. The p47^phox−/−^ coronary microvascular endothelial cells (CMEC) were isolated from the hearts of 10–12 week-old p47^phox^ KO mice as described previously (18, 27) and were used at passage 2. The gene transfection was performed exactly as described previously (18). After 48 h of gene transfection, cells were treated with vehicle, or with phorbol 12-myristate 13-acetate (PMA, Sigma) 100 ng/ml for 30 min to activate the NADPH oxidase. Cells were used immediately for ROS measurement and cell membrane isolation.

##### Preparation of Cell Membrane Fraction

The cell membrane protein fraction was prepared in MOPS-KOH buffer (MOPS-KOH 20 mmol/liter, sucrose 250 mmol/liter, pH 7.4) containing PMSF (1 mmol/liter), EDTA (0.1 mmol/liter), sodium fluoride (50 mmol/liter), sodium vanadate (2 mmol/liter), leupeptin (2 μmol/liter), and pepstatin (2 μmol/liter). The fractions of cell nuclei, mitochondria, submitochondria, and small organelles were eliminated by sequential ultracentrifugation as described previously (11), and the final pellet of membrane fraction was obtained after 60 min of centrifugation at 100,000 × *g*. Membrane proteins were analyzed for NADPH-dependent oxidase activity by lucigenin chemiluminescence or used for immunoblotting.

##### Immunoprecipitation and Immunoblotting

Immunoprecipitation of p47^phox^ was performed as described previously (18). Subsequent immunoblotting was performed using either phospho-serine specific monoclonal antibody for p47^phox^ phosphorylation or antibody to p22^phox^ for their association. For quantification of phos-p47^phox^ or p22^phox^ pulled-down with p47^phox^, the levels of total p47^phox^ detected in the same sample were used as loading controls.

##### Measurement of O_2_^⨪^ Production

The O_2_^⨪^ production was measured by lucigenin (5 μmol/liter)-chemiluminescence as described previously (28, 29). NADPH (100 μm) was added into the cell homogenates as substrate for the detection of NADPH-dependent O_2_^⨪^ production. The specificity of O_2_^⨪^ detection was confirmed by tiron (5 mmol/liter, an O_2_^⨪^ scavenger). Potential enzymatic sources of O_2_^⨪^ production were verified using inhibitors: rotenone (100 μmol/liter), oxypurinol (100 μmol/liter), or diphenyleneiodonium (DPI, 20 μmol/liter). As an independent approach, the intracellular ROS production by adherent cells was examined by 5-(and 6)-chloromethyl-2′,7′-dichlorodrofluorescein diacetate (DCF, Invitrogen) (28). Briefly, cells cultured onto chamber slides were incubated with 5 μmol/liter of DCF in Hanks' buffer for 15 min at 37 °C with or without PMA stimulation. DCF fluorescence at an excitatory wavelength of 495 nm was immediately acquired using Olympus BX61 fluorescence microscopy. Fluorescence intensity was quantified from at least 3 random fields (269.7 × 269.2 μm) per chamber, >1000 cells assessed per cell culture experiment, and at least 3 separated cell cultures per condition. Tiron (10 mmol/liter), a cell membrane permeable non-enzymatic scavenger of O_2_^⨪^ was used to verify the detection of O_2_^⨪^ (29).

##### Fluorescence Microscopy

Immunofluorescence microscopy was performed as described previously (18). Antibody binding was detected by extravidin-FITC (green) or streptavidin-Cy3 (red). Normal rabbit or goat IgG (5 μg/ml) were used instead of primary antibody as negative controls. Images were acquired with an Olympus BX61 fluorescence microscope system as described previously (30).

##### Statistics

For the *in vitro* studies, data were presented as means±S.D. of results taken from at least 3 independent cell cultures/per condition. In the case of CMEC, each isolation used 6 mice/per group, and the data presented were the mean from at least 3 isolations. Comparisons were made by one-way ANOVA with Bonferroni test analysis, and *p* < 0.05 was considered statistically significant.

## RESULTS

### 

#### 

##### Computer Structural Model of Full p47^phox^ Protein

**FIGURE 1.**
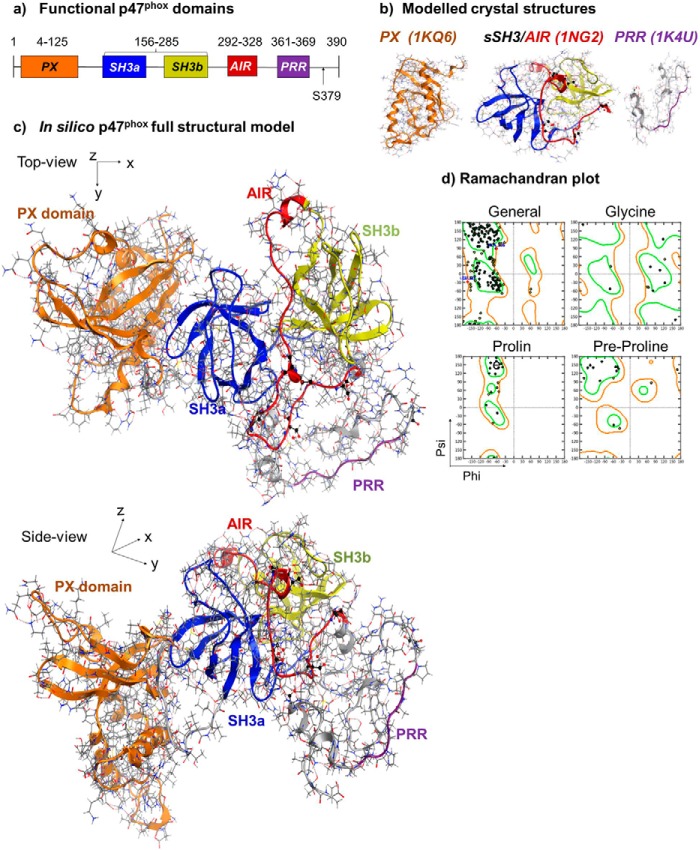
**Computer model of the complete p47^phox^ protein structure.** *a*, illustration of p47^phox^ functional domains. *b*, computer models of the crystal structures of p47^phox^ functional domains shown in skeleton depiction with overlaid *ribbons. Left panel*: PX domain (*orange color*). *Middle panel*: Auto-inhibited sSH3 and AIR: SH3a (*blue*), SH3b (*yellow*) and AIR (*red*). *Right panel*: C-terminal tail (*gray*) with PRR (*purple*). *c*, computer model of complete autoinhibited p47^phox^ with functional domains in the same color scheme as in *b. d*, representative Ramachandran plot for structural analysis of the p47^phox^ protein model in autoinhibited form.

To produce a reliable structural model of the full p47^phox^ protein, we initially generated computational models from three available crystal structures of the p47^phox^ functional domains: the PX-domain (a.a 1–141, 1KQ6), the sSH3/AIR-domain (a.a. 159–340, 1NG2), and the PRR domain (a.a. 359–390, 1K4U) (Fig. 1, *a* and *b*). Two missing linking peptides (142-MKDGKSTATDITGPII-156 and 340-PGPQSPGSPLEEERQTQRSK-360) between these structures were generated using the homology modeling server Phyre2 with a 50 and 90% structural confidence, respectively. A full p47^phox^ structure of the auto-inhibited form was generated using the *in silico* homology modeling technique (Fig. 1*c*). The structure has no distorted torsion angles or residues in disallowed regions as analyzed using a Ramachandran plot (Fig. 1*d*), and all internal hydrogen-bonds were optimally occupied as an independent measure of structural reliability. Furthermore, the generated p47^phox^ structure is extended rather than globular, with a radius of gyration in the same order of magnitude that was published previously in a study employing small angle x-ray scattering (31).

##### Molecular Dynamics (MD) Simulation and p47^phox^ Phosphorylation-induced Conformational Changes

**FIGURE 2.**
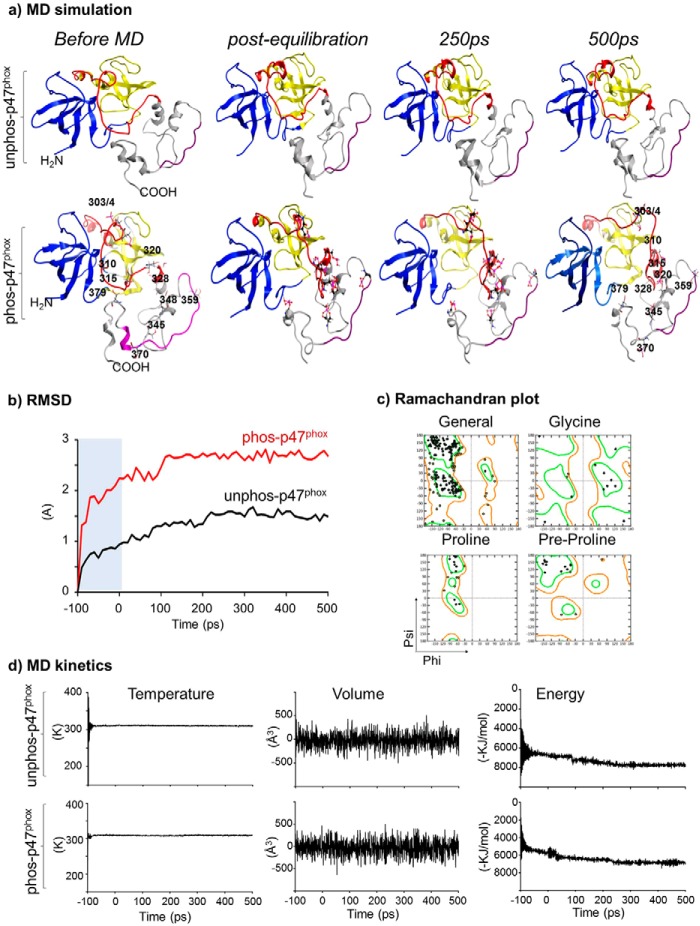
**MD simulation of the p47^phox^ structure containing sSH3, AIR, and C-terminal tail.** *a*, structural changes of p47^phox^ shown in ribbon depiction. *Upper panel*: unphosphorylated form. *Lower panel:* phosphorylated form. Phospho-serines at 303, 304, 310, 315, 320, 328, 345, 348, 359, 370, and 379 are shown in *pink skeleton representation. b*, kinetic changes of the RMSD over the time course of MD simulation in comparison to their respective pre-MD structures. *Shaded region* denotes the pre-equilibration stage of the simulation. *c*, representative Ramachandran plot of phosphorylated p47^phox^ structure at 500 ps of MD for structural reliability. *d*, MD simulation temperature (*left panels*), volume (*middle panels*), and energy (*right panels*) of both unphosphorylated (*upper panel*) and phosphorylated p47^phox^ (*bottom panel*).

To examine the conformational changes in the p47^phox^ structure toward the C terminus, the PX domain was omitted, and the rest of the structure (a.a. 156–390) was subjected to MD simulation (Fig. 2). We found that without phosphorylation, the auto-inhibited p47^phox^ model remained stable over the MD time course (post-equilibration, 250 ps and 500 ps of MD) (Fig. 2*a*, *upper panel*). Importantly, there were no noticeable core structural changes between the pre-MD and after MD with a RMSD of the α-carbon backbone being 1.493 Å (Fig. 2*b*). The lack of gross structural changes throughout the MD simulation confirms the stability of the generated p47^phox^ model used in this study.

We then added a phosphate (PO_4_^3−^) group to the side-chains of Ser-303, Ser-304, Ser-310, Ser-315, Ser-320, Ser-328, Ser-345, Ser-348, Ser-359, Ser-370, and Ser-379 (Fig. 2*a*, *lower panel*). The addition of phosphate groups before MD had no significant effect on the core structural stability of the p47^phox^ because the radius of convergence of molecular mechanics used in this way was quite small and the local structural energy was accordingly minimized to accommodate the changes to keep the structural stability. The sSH3 remained completely masked by the AIR (Fig. 2*a*, *lower panel*, *left image*). However, when the structure was subjected to MD simulation, the interaction between the C-terminal tail, AIR, and sSH3 underwent rapid dissociation that released AIR in less than 100 ps of MD. There was a time-dependent sequential structural change of the AIR during the course of MD simulation with the movement of the phosphorylated double serines (Ser-303/4) as the final step to fully open the sSH3 groove (Fig. 2*a*, *lower panel* at 500 ps). Compared with the un-phosphorylated structure, the p47^phox^ α-carbon backbone RMSD kinetics in the phosphorylated form increased rapidly during the equilibration period (<100 ps) and plateaued afterward during the MD time course (Fig. 2*b*). The phosphorylated p47^phox^ structure after MD had no distorted torsion angles or residues in disallowed regions as analyzed using a Ramachandran plot (Fig. 2*c*). There was no significant difference in the experimental settings of unphosphorylated and phosphorylated forms in terms of MD simulation parameters, *i.e.* temperature and volume (Fig. 2*d*).

##### Crucial Role of Ser-379 Phosphorylation in Initiating AIR Instability and p47^phox^ Conformational Change

**FIGURE 3.**
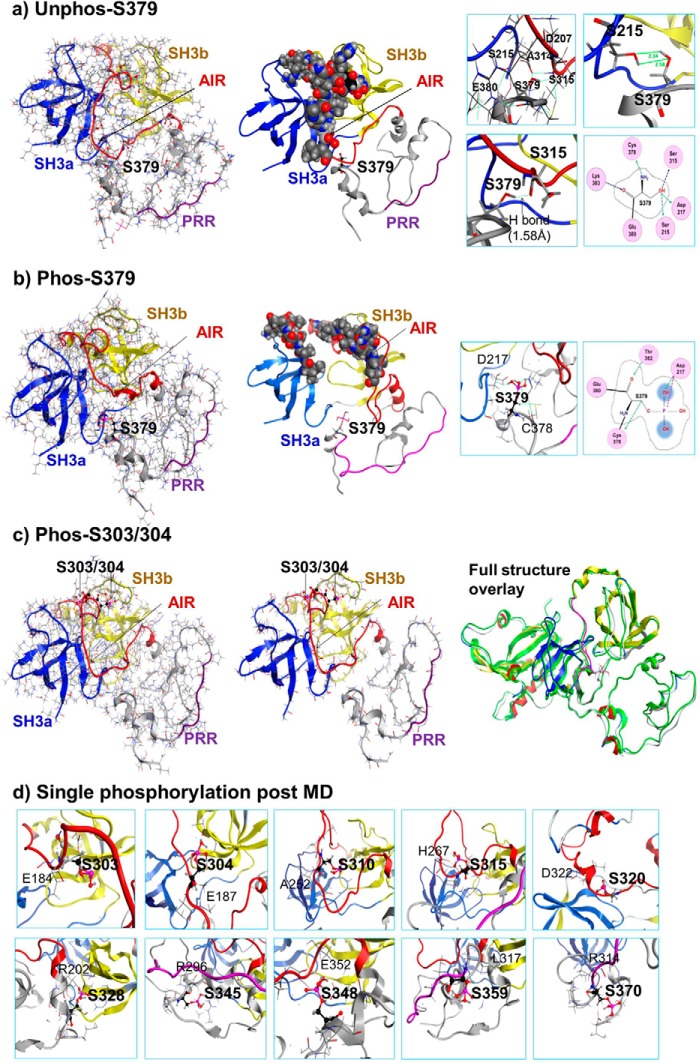
**Structure analysis of single serine phosphorylation at post-equilibration and at 500 ps of MD simulation.** Phosphorylated serine is shown in *pink skeleton. a*, unphosphorylated Ser-379 in ribbon overlaid skeleton structure. *Left panel:* post-equilibration. *Middle panel:* post-MD (500ps). AIR in the sSH3 groove is presented in *atomic volume balls. Right panel*: Ser-379 local residue interaction maps. Hydrogen bonds are shown in *green* with distance labeled. *b*, phosphorylated Ser-379. *Left panel:* post-equilibration. *Middle panel:* post-MD (500 ps). *Right panel*: phospho-Ser-379 local residue interaction maps. Hydrogen bonds are shown in *green. c*, phosphorylated Ser-303/4. *Left panel:* post-equilibration. *Middle panel:* post-MD (500 ps). *Right panel*: Superposed images of p47^phox^ unphosphorylated (in *green color*) and Ser-303/4 phosphorylated at 500 ps of MD (same image as in the *middle panel*). *d*, local residue interaction maps of single individual serine phosphorylation (as indicated in the map) at 500 ps of MD.

Our MD data (Fig. 2*a*) revealed an unexpected discovery that the movement of the phosphorylated C-terminal tail occurred before AIR destabilization. There are at least three known phosphorylation sites of PKC (Ser-359, Ser-370, and Ser-379) located in p47^phox^ C-terminal tail. The phosphorylation of Ser-359 and Ser-370 had been found to have no significant effect on p47^phox^ interaction with other phox subunits (16). However, the phosphorylation of Ser-379 was found to be essential for TNFα-induced ROS production by NADPH oxidase and redox-signaling in endothelial cells, although the molecular mechanism remained unknown (32). To find out how a single Ser-379 phosphorylation could affect p47^phox^ function, we performed *in silico* single Ser-379 phosphorylation and MD simulation. Before phosphorylation, Ser-379 was in close proximity to both sSH3 and the AIR (Fig. 3*a*), and formed a H-bond with Ser-215 located in the hinge region of sSH3 and another H-bond between Ser-379 side chain and the main chain of Ser-315 located in the central part of the AIR, which tied AIR into the tandem SH3 groove (Fig. 3*a*, residue interaction images in the right panel), MD simulation (500 ps) of un-phosphorylated Ser-379 had no significant effect on the autoinhibited structure of p47^phox^ (Fig. 3*a*, *middle panel*). When Ser-379 was phosphorylated and subjected to MD simulation, the negative charge generated broke the previous links immediately during the equilibration period and caused the C-terminal tail to move (Fig. 3*A*, *left panel*). After 500 ps of MD simulation, new H-bonds were formed between phosphorylated Ser-379 and Thr-382 located in the C-terminal tail and between the phosphate group of Ser-379 and the Asp-217 as a way to neutralize the local environment charges (Fig. 3*c*, *right panel*). The AIR was free, and the sSH3 groove was partially exposed (Fig. 3*b*, *middle panel*).

Although our data strongly indicate that Ser-379 phosphorylation is an early key event in p47^phox^ activation, we need to confirm if it is also linked with any other single serine phosphorylation. The p47^phox^ has a double serine phosphorylation site (Ser-303/4) located within the AIR, therefore, we checked firstly the Ser-303/4 phosphorylation. There was no significant structural change associated with Ser-303/4 phosphorylation at post-equilibration (Fig. 3*c*, *left panel*) and at 500 ps of MD (Fig. 3*c*, *middle panel*). Overlapping images of the unphosphorylated and phosphorylated structures overlaid further confirmed no significant structural change (Fig. 3*c*, *right panel*). We then examined one by one the single phosphorylation plus 500 ps of MD of Ser-303, Ser-304, Ser-310, Ser-315, Ser-328, Ser-345, Ser-348, Ser-359, and Ser-370, and confirmed that there was no significant structural change associated with these single serine phosphorylations. The local residue interaction maps taken after phosphorylation and 500ps of MD (Fig. 3*d*) remained the same as the maps taken before phosphorylation.

##### Interaction Kinetics of Phosphorylated p47^phox^ with p22^phox^

**FIGURE 4.**
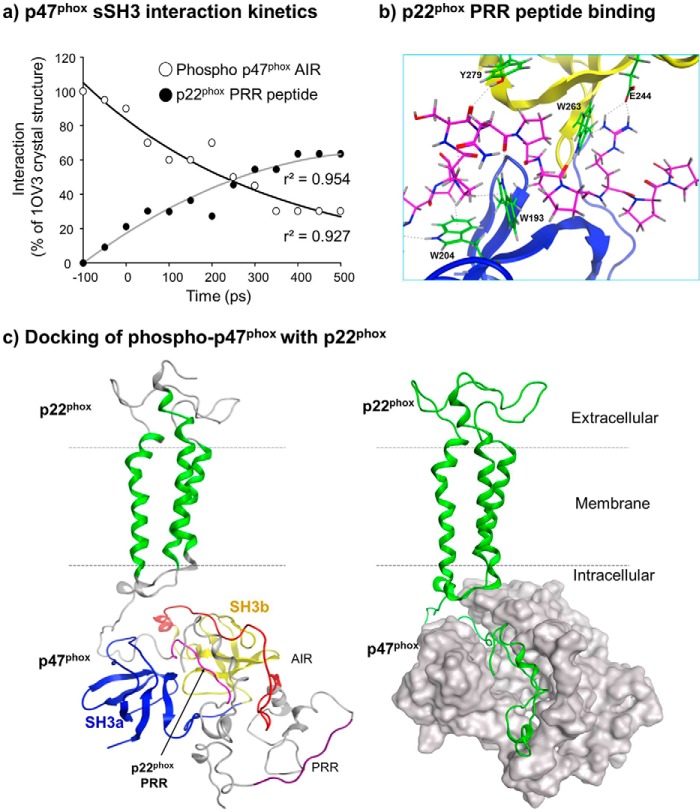
**Interaction between phosphorylated p47^phox^ and p22^phox^.** *a*, interaction kinetics of p47^phox^ sSH3 with AIR (*open circles*) or with p22^phox^ PRR peptide (*filled circles*) during MD simulation. *b*, interactions of both p47^phox^ SHa (*blue*) and SHb (*yellow*) with p22^phox^-PRR peptide (*pink skeleton*). *Green skeletons* represent p47^phox^ residues interacting with p22^phox^ PRR peptide. *c*, models of global structural interactions between p47^phox^ and p22^phox^ following MD simulation. *Left panel:* Docking of phosphorylated p47^phox^ and p22^phox^ in ribbon presentation. p22^phox^ PRR is in *pink color. Right panel:* docking of p47^phox^ (*silver space-filled* presentation) with p22^phox^ (*green*).

It is well documented that the cytosolic subunits of the NADPH oxidase anchor at membranes through an interaction between the p47^phox^ sSH3 groove and p22^phox^ PRR (33). However due to the lack of the entire protein structure, the interaction dynamics between p47^phox^ phosphorylation, AIR dissociation, and binding to p22^phox^ remains unclear. Using a MD-based docking technique we examined the interaction kinetics of phosphorylation-induced dissociation between AIR and sSH3 of the p47^phox^ and the binding of p47^phox^ sSH3 with the p22^phox^ PRR peptide (sequence: QPPSNPPPRPP) (Fig. 4, *a* and *b*). We found a time-dependent decrease in the intramolecular interactions between the p47^phox^ AIR and sSH3 (r^2^ = 0.954), and this was accompanied with a time-dependent increase in the interaction between the p22^phox^ PRR peptide and the p47^phox^ sSH3 (r^2^ = 0.927) (Fig. 4*a*). At 500 ps of MD simulation, the p22^phox^ peptide migrated completely into the p47^phox^ sSH3 binding groove in contact with the key p47^phox^ residues as shown in the crystallized structure (1OV3) (14) except for the residue of Asp-243 (Fig. 4*B*). We also examined the protein-protein interaction between our phosphorylated p47^phox^ model and the consensus p22^phox^ model published previously (25) (Fig. 4*C*). We found that both the p22^phox^ and p47^phox^ models remained stable throughout the MD simulations and bound together successfully through the interaction between p47^phox^ sSH3 and p22^phox^ PRR to form a p47^phox^/p22^phox^ protein complex, which for the first time provides mechanistic insight into the global association of these two proteins.

##### The Effect of Single p47^phox^ Serine to Alanine Mutation on Acute PMA Stimulation-induced O_2_^⨪^ Production by p47^phox−/−^ Endothelial Cells after Gene Transfection

**FIGURE 5.**
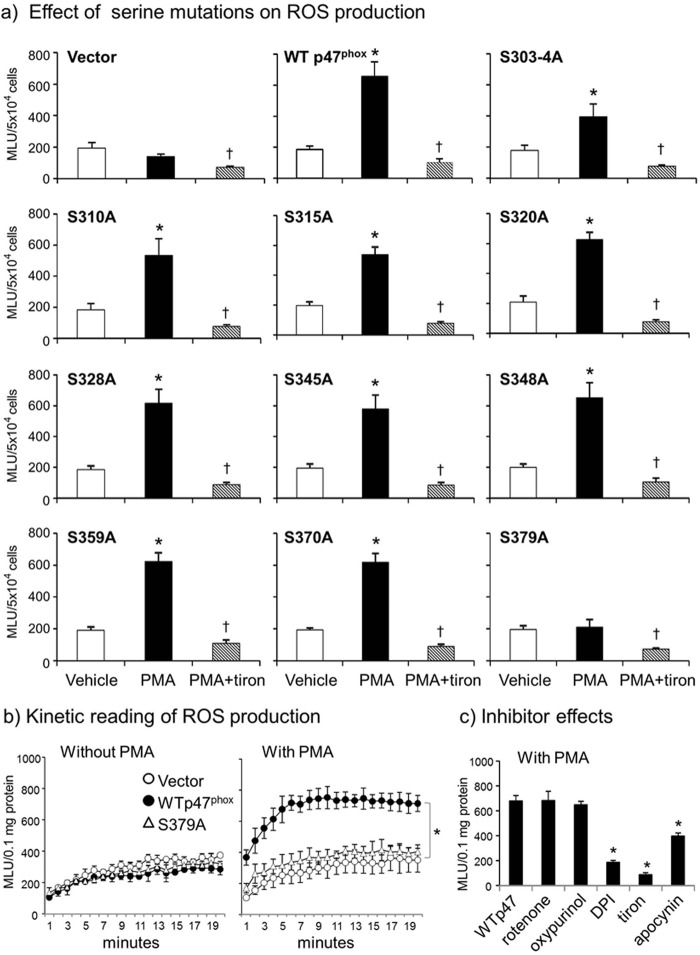
**The effect of serine to alanine mutations on PMA-induced O_2_^⨪^ production by p47^phox−/−^ CMEC after gene transfection.** NADPH-dependent O_2_^⨪^ production was detected by lucigenin (5 μm) chemiluminescence in cell homogenates. *a*, individual serine to alanine mutations. *, *p* < 0.05 for PMA values *versus* vehicle values in the same group. †, *p* < 0.05 for indicated values *versus* PMA values in the same group. *n* = 3 independent CMEC isolations and gene transfection experiments. *b*, S379A mutation. *Left panel:* kinetic measurements of basal O_2_^⨪^ production (without PMA stimulation); *right panel:* kinetic measurements of PMA-stimulated O_2_^⨪^ production; *c,* effects of different enzyme inhibitors on PMA-stimulated O_2_^⨪^ production. *, *p* < 0.05 for indicated values *versus* WT p47^phox^ cDNA transfected value in the same panel.

To confirm the *in silico* findings where Ser-379 phosphorylation is shown to be crucial for p47^phox^ activation, we performed site-directed mutagenesis by replacing eleven serines individually in the human p47^phox^ cDNA with alanine which could not be phosphorylated. An empty vector (PcDNA3.1/Zeo) was used as a negative control and the wild-type p47^phox^ cDNA was used as a positive control. Mutated and wild-type p47^phox^ cDNA constructs were used to transfect the primary CMEC isolated from the p47^phox−/−^ mice. The cells were then stimulated with either vehicle or a potent protein kinase C activator, PMA (100 ng/ml, for 30 min) to induce p47^phox^ phosphorylation, and then examined for NADPH oxidase activity by detecting the levels of NADPH-dependent O_2_^⨪^ production (Fig. 5*a*). Tiron (an O_2_^⨪^ scavenger) was used to confirm the assay specificity. We found that there was no significant difference in the basal (without PMA) levels of O_2_^⨪^ production by p47^phox−/−^ cells after gene transfection with different constructs. However, when the cells were stimulated with PMA and compared with cells transfected with an empty vector, cells transfected with any constructs *i.e.* wild-type (WT) p47^phox^ cDNA, S303–4A, S310A, S315A, S320A, S328A, S345A, S349A, S359A, and S370A increased significantly the levels of O_2_^⨪^ production, except the cells transfected with S379A. It was obvious that Ser-379 phosphorylation had a key role in initiating p47^phox^ phosphorylation-dependent O_2_^⨪^ production by the NADPH oxidase.

**FIGURE 6.**
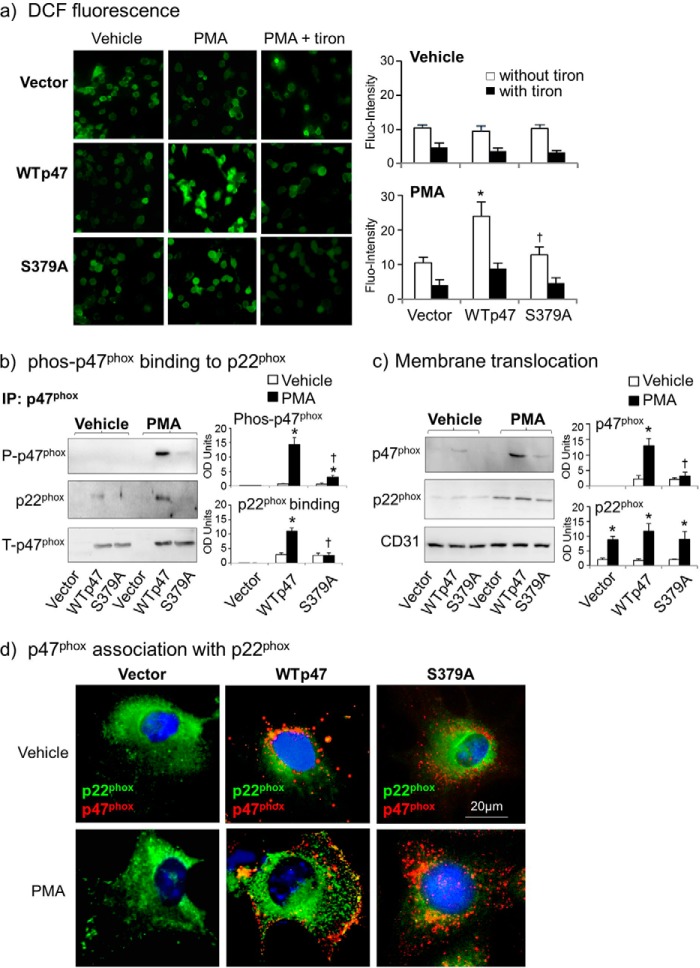
**Effects of S379A mutation on PMA-induced cell ROS production, p47^phox^ binding to p22^phox^ and membrane translocation in p47^phox−/−^ CMEC after gene-transfection.** WTp47 represents wild-type p47^phox^ cDNA. *a*, DCF detection of intracellular ROS production by intact cells. *, *p* < 0.05 for indicated values *versus* vector values in the same group. †, *p* < 0.05 for indicated values *versus* WTp47 values in the same treatment group. *n* = 3 independent CMEC isolations and gene transfection experiments. *b*, p47^phox^ was pulled down by antibody-bound beads (*IP*) and detected by Western blot for the levels of p47^phox^ serine phosphorylation and binding to p22^phox^. The optical densities (*OD*) of protein bands were quantified digitally and normalized to the levels of total p47^phox^ detected in the same samples. *c*, CMEC membrane fractions were detected for the levels of expression of p47^phox^ and p22^phox^. The OD of protein bands were quantified digitally and normalized to the levels of CD31 detected in the same samples. *, *p* < 0.05 for PMA values *versus* vehicle values in the same group. †, *p* < 0.05 for indicated values *versus* PMA values of WT p47^phox^ cDNA-transfected cells. *n* = 3 independent CMEC isolations and gene transfection experiments. *d*, fluorescence microscopic images of p47^phox−/−^ CMEC after gene transfection. The p47^phox^ is in *red color* and p22^phox^ is in *green color*, the *yellow* fluorescence indicates the co-localization of p47^phox^ with p22^phox^.

The effect of S379A on acute PMA-induced O_2_^⨪^ production by endothelial cells was further investigated in detail (Fig. 5*b*). Without PMA stimulation, there was no significant difference in the basal levels of O_2_^⨪^ production between cells transfected with an empty vector and cells transfected with wild-type p47^phox^ cDNA or with S379A mutated construct (Fig. 5*b*, *left panels*). However, when the cells were stimulated with PMA, WT p47^phox^ cDNA transfected cells significantly increased O_2_^⨪^ production, which was completely inhibited back to the vector control levels in S379A-transfected cells (*p* < 0.05) (Fig. 5*b*, *middle panel*). The enzymatic source of PMA-induced O_2_^⨪^ production by WT p47^phox^ cDNA transfected cells was examined using different enzyme inhibitors. It was abolished by tiron, an O_2_^⨪^ scavenger, significantly inhibited by DPI (a flavo-protein inhibitor) and apocynin (a NADPH oxidase inhibitor), but not by inhibitors to the mitochondrial complex-1 enzymes (rotenone) or to xanthine oxidase (oxypurinol) (Fig. 5*b*, *right panel*). As an independent approach, the effect of S379A on PMA-induced O_2_^⨪^ production by intact adherent CMEC was double confirmed by tiron-inhibitable DCF fluorescence (Fig. 6*a*).

##### The Effects of S379A Mutation on PMA-induced p47^phox^ Phosphorylation, Membrane Translocation, and Binding to p22^phox^ in Cells

To confirm if Ser-379 phosphorylation was indeed required for the chain reaction of PMA-induced p47^phox^ phosphorylation, membrane translocation, and complex formation with p22^phox^, we immune-pulled-down (IP) total p47^phox^ protein and detected for the levels of serine phosphorylation and the levels of p22^phox^ pulled-down together with p47^phox^ (Fig. 6*b*). In vehicle-stimulated cells, the levels of p47^phox^ phosphorylation and binding to p22^phox^ were not detected in vector-transfected cells, and were just detectable in cells transfected with wild-type p47^phox^ or S379A. However, when the cells were stimulated with PMA, WT p47^phox^ cDNA-transfected cells significantly increased the levels of p47^phox^ phosphorylation and the association with p22^phox^, and these were inhibited back to vector-transfected control levels in S379A construct transfected cells.

The p47^phox^ membrane translocation was examined in membrane fractions isolated from p47^phox−/−^ CMEC after gene transfection (Fig. 6*c*). The p47^phox^ was absent in the membrane fraction of vector transfected cells. After PMA stimulation, p47^phox^ was highly detected in the membrane fraction of WT p47^phox^ cDNA transfected cells, and this was significantly reduced in S379A-transfected cells (Fig. 6*c*). The inhibitory effect of S379A on p47^phox^ membrane translocation and association with p22^phox^ was further confirmed by immunofluorescence (Fig. 6*d*). The p47^phox^ was labeled by a goat polyclonal antibody and detected by Cy3 (red color), and p22^phox^ was labeled by a rabbit polyclonal antibody and detected by FITC (green color). In vector-transfected cells, p22^phox^ was detected across the entire cells. PMA stimulation induced p22^phox^ clustering and membrane localization. The p47^phox^ was detected in wild-type p47^phox^ cDNA and S379A construct transfected cells. PMA stimulation induced p47^phox^ plasma membrane translocation and association with p22^phox^ as indicated by the yellow fluorescence seen only in WT p47^phox^ cDNA-transfected cells, but not in S379A mutation transfected cells.

## DISCUSSION

Rapid NADPH oxidase activation in neutrophils is crucial for immune response to pathogen invasion (16, 17). However inappropriate activation of NADPH oxidase causes inflammation and oxidative damage to cells with severe consequences (1). Insight into the mechanism of NADPH oxidase activation is important for new drug development and disease prevention. p47^phox^ phosphorylation has been recognized as a prerequisite for NADPH oxidase assembly and activation (14, 15, 34, 35). However, due to the lack of complete protein three-dimensional structural information, the molecular mechanisms that govern the p47^phox^ phosphorylation cascade, assembly with cytochrome *b558* and O_2_^⨪^ production by NADPH oxidase remain unclear. In the current study we have, for the first time, presented a complete *in silico* model of the full p47^phox^ protein structure in both auto-inhibited and activated forms. Our structural model is generated by a combination of the existing x-ray crystal structures of p47^phox^ functional domains, and is stable under MD simulation. There is no evidence of disordered contacts or residues in our model as shown by the Ramachandran plot, and internal hydrogen bonds were optimally occupied, which is an independent measure of structural reliability. Our model provides a powerful tool for further investigation of the function of the p47^phox^ and the NADPH oxidase.

Although crystal structures of several functional domains of p47^phox^ and their role in p47^phox^ activation had been reported previously (14–16, 36, 37), these structures remain as disconnected pieces and none of these previous studies had examined the location and a possible role of p47^phox^ C-terminal tail in p47^phox^ activation. A peptide of the p47^phox^ C-terminal tail was reported previously to bind to the SH3 pocket of other phox proteins regardless of phosphorylation (19). The novelty of the current study is that it not only provides a structural model of the full p47^phox^ protein but also revealed an important mechanism as to how these functional domains *i.e.* sSH3, AIR, and C-terminal tail interact with each other and regulate p47^phox^ function in its three-dimensional form. Using a combination of *in silico* phosphorylation, MD simulation, and protein/protein docking we demonstrated for the first time that the C-terminal tail plays a key role in connecting AIR to sSH3 and maintaining p47^phox^ structural stability at rest and in the regulation of p47^phox^ activation. During the time course of phosphorylation and MD simulation, there was a swift disconnection (<100 ps of MD) between the C-terminal tail and the AIR and the sSH3, and the movement of the phosphorylated C-terminal tail occurred before the AIR phosphorylation. The entire AIR phosphorylation process was a well organized chain reaction with the movement of phosphorylated double serine, Ser-303/4 as the final step to fully open the sSH3 binding site. This is important novel information on p47^phox^ regulation, and may have a broad application to other SH3 domain-containing proteins.

The p47^phox^ has multiple serine phosphorylation sites within and outside the AIR toward its C terminus. Previously we only knew that extensive p47^phox^ phosphorylation of AIR is required for p47^phox^ activation, but we did not know the molecular switch of the AIR phosphorylation cascade. An important contribution of the current study is that we have discovered Ser-379 phosphorylation as a molecular switch to initiate the AIR phosphorylation cascade. At rest, unphosphorylated Ser-379 forms H-bonds with the hinge of sSH3 and the AIR, which connects the sSH3 and AIR together and stabilizes p47^phox^ in its autoinhibited form. When the Ser-379 is phosphorylated, it disrupts the links with the sSH3 and AIR, and in turn frees AIR for subsequent phosphorylation. The unique role of Ser-379 phosphorylation as a molecular switch in p47^phox^ activation has been further confirmed by extensive experimental approaches of single serine phosphorylation and MD simulation one by one of eleven potential phosphorylation sites. It is clear that none of the other single serine phosphorylation sites or even the double serine phosphorylation sites (Ser-303/4) can cause significant conformational change of AIR.

Previously, the p22^phox^ was reported to interact with the p47^phox^ N-terminal SH3a domain rather than the C-terminal SH3b domain (37). In this study we found that both SH3 domains interact with p22^phox^ PRR. We have presented successfully a global complex structure model through the protein/protein docking of the p22^phox^ PRR and the sSH3 of p47^phox^ protein, which for the first time provides a conformational model of the two phox proteins together. The ability to decipher the molecular opening of the p47^phox^ binding site to p22^phox^ is crucial for specific inhibitor design that control partial or full activation of NADPH oxidase in different cell types. Endothelial cell NADPH oxidase shares the same molecular structure with neutrophil NADPH oxidase. However, NADPH oxidase in resting endothelial cells is already partially activated (34, 38). Insight into how NADPH oxidase activity is differentially regulated in different cell types is important for novel drug discovery to treat oxidative stress-related diseases without affecting the neutrophil oxidative burst response to pathogen invasion. Our three-dimensional structural model of the entire p47^phox^ protein and models of step by step phosphorylation dynamics fill the gap in current Nox research and provide valuable insight for future investigation.

The *in silico* discovery was further confirmed by site-directed mutagenesis and gene transfection of the p47^phox^ knock-out CMEC. By acute stimulation of cells with PMA, a powerful pan-PKC activator, we showed that 30 min stimulation by PMA was able to induce significant O_2_^⨪^ production in cells transfected with any of the serine to alanine mutated constructs in p47^phox^ protein, except the S379A mutation. S379A mutation completely blocked acute PMA stimulation-induced: (i) p47^phox^ binding to p22^phox^, as evidenced by immuno-pull-down assay and Western blot; (ii) p47^phox^ membrane translocation, as evidenced by cell membrane fractionation followed by Western blot and immunofluorescence; and (iii) O_2_^⨪^ production by NADPH oxidase, as examined by both lucigenin-chemiluminescence in cell homogenates and DCF fluorescence in intact cells. These *in vitro* data provide strong evidence in support of our *in silico* models that Ser-379 phosphorylation is a molecular switch of the p47^phox^ phosphorylation cascade in response to agonist stimulation.

In summary, we have presented for the first time an *in silico* three-dimensional model of the entire p47^phox^ protein and revealed an important mechanism of p47^phox^ activation. The molecular insight of how Ser-379 phosphorylation in the C-terminal tail initiates p47^phox^ activation and how phosphorylation dynamically controls partial or full opening of the p47^phox^ binding site can be further exploited for developing specific NADPH oxidase inhibitors to treat oxidative stress-related diseases.

## Footnotes

2 The abbreviations used are:

ROS: reactive oxygen species
AIR: autoinhibitory region
CMEC: coronary microvascular endothelial cells
DCF: 5-(and 6)-chloromethyl-2′,7′-dichlorodrofluorescein diacetate
DPI: diphenyleneiodonium
KO: knockout
MD: molecular dynamics
MOE: molecular operating environment
PMA: phorbol 12-myristate 13-acetate
PRR: proline-rich region
ps: picoseconds
RMSD: root mean squared deviation
SH3: Src homology 3
sSH3: super-SH3.
